# Loneliness, Social Integration and Consumption of Sugar-Containing Beverages: Testing the Social Baseline Theory

**DOI:** 10.1371/journal.pone.0104421

**Published:** 2014-08-08

**Authors:** Roger Ekeberg Henriksen, Torbjørn Torsheim, Frode Thuen

**Affiliations:** 1 Centre for Evidence-Based Practice, Bergen University College, Bergen, Norway; 2 Faculty of Psychology, Department of Psychosocial Science, University of Bergen, Bergen, Norway; Faculty of Biology, Spain

## Abstract

**Objective:**

Social Baseline Theory (SBT) proposes that close relationships aid in metabolic resource management and that individuals without significant relationships may experience more demands on their own neural metabolic resources on a daily basis when solving problems, remaining vigilant against potential threats and regulating emotional responses. This study tests a hypothesised consequence derived from SBT: relative social isolation leads to increased levels of sugar intake.

**Methods:**

Based on cross-sectional, self-reported data from the Norwegian Mother and Child Cohort Study (N = 90 084), information on social integration and the consumption of both sugar-sweetened and artificially sweetened sodas and juices was obtained from a large number of women in early pregnancy. Multiple regression analyses were conducted to assess whether loneliness, marital status, relationship satisfaction, advice from others than partner, and cohesion at work is associated with consumption of sodas and juices.

**Results:**

Perceived loneliness was associated with elevated intake of all sugary beverages, while relationship satisfaction was negatively associated with all sugary beverages. Being married or cohabitating, having supportive friends, and having a sense of togetherness at work were associated with lower intake of two out of three sugar-containing beverages. These associations were significant, even after controlling for factors such as body mass index, weight related self-image, depression, physical activity, educational level, age and income. In comparison, a statistically significant relationship emerged between relationship satisfaction and artificially sweetened cola. No other predictor variables were significantly associated with any type of artificially sweetened beverage.

**Conclusions:**

This study indicates that loneliness and social integration influence the level of consumption of sugary beverages. The results support the hypothesis derived from the Social Baseline Theory that relative social isolation leads to increased levels of sugar intake.

## Introduction

This study examines whether there is an association between social integration and sugar intake, as proposed by the Social Baseline Theory [1].

The brain is the most metabolically active organ of the human body. Even in a resting state of non-specific neural activity, it counts for as much as 20% of the body's total energy utilization [2]. The adult human brain is almost completely dependent on glucose as a source for energy. Unlike other organs, the brain has very little metabolic reserve because it oxidizes glucose at relatively close to its maximum rate. Because more than 85% of the glucose is utilized by neurons, the rate at which the brain oxidizes glucose is proportional to neuronal activity [2], [3]. This implies that specific neural effort must be followed by a rapid supply of glucose from the blood in an amount that corresponds with the metabolic cost of the specific effort [3]. In an illustrative study, Scholey, Harper and Kennedy [4] showed that a cognitively demanding subtraction task was followed by an immediate, significant fall in peripheral blood glucose. Moreover, by offering a glucose-containing drink to one group and a placebo drink to the other, they also demonstrated that glucose intake after a fall in blood glucose level was associated with better performance on a subsequent cognitively demanding task. Similar studies were performed by Gailliot and colleagues [5], who found that different types of mental effort, such as thought suppression, attention control and emotion regulation, were followed by an immediate fall in peripheral blood glucose levels.

Restoring the blood glucose to normal levels after a drop is of high biological priority. Even a few seconds without cerebral glucose oxidation will lead to unconsciousness, and after a few minutes, the death of neurons will occur. In order to counteract a dangerous glucose shortage in the brain, humans possess a highly sophisticated glucose regulatory system [6]. In addition to the role of hepatic glycogen in counter-regulation of hypoglycemia [7], the brain has the ability to rapidly sense a drop in circulating glucose and induce a range of other counter-regulatory responses. These include the secretion of galanin and GABA, which minimizes the chance of glucose deprivation by inhibiting neural activity, as well as stimulating cortisol release, which aims to increase blood sugar through gluconeogenesis in the liver [2], [6]. In addition, cortisol has been shown to suppress insulin secretion from pancreatic b-cells and impair insulin initiated translocation of the intracellular glucose transporter, GLUT 4 [8]–[10]. This leads to decreased insulin-dependent glucose uptake into peripheral cells and makes glucose available to the brain via glucose transporter GLUT 1 [3], [10], [11]. Another set of regulatory mechanisms involves directed behavior. Naturally, most efficient are behavioral actions that regulate glucose levels by facilitating exogenous supplies of carbohydrates from nutritional sources. Indeed, a substantial body of research has linked different types of demanding mental work to the subsequent intake of food, with a significant preference for foods high in carbohydrates [12]–[18]. This point is thoroughly discussed by Peters, Kubera, Hubold and Langemann [19] who have developed a model called the Cerebral Supply-Chain Model, which explains how both cortisol-induced and eating-induced supplies of glucose are initiated by the brain during times of energy-demanding psychosocial stress.

During periods of evolutionary pressure, it is likely that individuals with efficient glucose regulatory responses have been evolutionarily favored. Social Baseline Theory (SBT) [1], [20], [21] argues that in order to conserve metabolic resources, the human brain is phylogenetically designed to benefit from social networks when engaging in demanding mental activities, such as effortful decision making, creative problem solving and emotion regulation. SBT uses the principles of risk distribution and load sharing to explain the brain's metabolic benefit from social interaction. *Risk distribution* refers to how individuals in a group perceive and react to environmental risk, as opposed to how the corresponding risk would be handled by socially isolated individuals. For example, as a group has more eyes and ears than a single individual, effort toward vigilance against possible dangers in the environment are distributed across the group. In this way, fewer neural resources are needed for each group member as compared with the cost of the precautions and worries solitary individuals must engage in to ensure a similar safety level. In close relationships, participants receive benefits beyond those associated with risk distribution. *Load sharing* refers to the energy-conserving effects of trust and interdependence, which are found between, for example, romantic partners, family members and close friends. In close relationships, the members tend to engage in behaviors that facilitate one another's well-being, for example, sharing resources, sharing goals and assisting in problem solving [22]. Central to SBT is the notion that “as an individual becomes accustomed to load sharing, his social system becomes an extension of the way his brain interacts with the world” [1]. The theory proposes that such “outsourcing” of mental work is particularly beneficial in the regulation of emotional responses because self-regulating processes supported by the prefrontal cortex are believed to be more metabolically costly than most other cognitive processes. Thus, socially mediated forms of emotion regulation may have significant energy-conserving advantages [1], [23], [24]. According to SBT, due to the evolutionary benefits of adjusting to the principles of risk distribution and load sharing, the human brain leans on social resources as a default strategy: “the human brain is designed to assume that it is embedded within a relatively predictable social network characterized by familiarity, joint attention, shared goals, and interdependence” [1]. A violation of this “baseline assumption”, however, signals the need for increased threat-related vigilance and reactivity. Thus, socially isolated individuals regulate their emotions at a higher metabolic expense compared with socially integrated individuals. In sum, SBT predicts that relative social isolation requires more neural metabolic resources in order to both manage daily life routines and to regulate emotions. This hypothesis builds on research showing that neural systems that support self-regulatory efforts are less active when social support is provided [20], [24]. Furthermore, in a context in which social support is provided, the down-regulation of neural response is a function of relational quality. In an fMRI-study conducted by Coan, Schaefer and Davidson [25], 16 women were confronted with the threat of a mild electric shock while either alone, holding a stranger's hand, or holding their partners' hands. While holding their partners' hands, the women in the highest-quality relationships showed the least threat-related brain activity. When holding a stranger's hand, the level of threat-related activity increased as compared with holding the partners' hands. Finally, when exposed to the threat of shock without the support of handholding, the threat-related brain activity reached its highest level.

Given that socially isolated individuals frequently demand extra supplies of glucose from the blood to maintain optimal brain function, one might expect elevated intake of food and beverages that rapidly elevate blood glucose in this group [19]. This assumption is consistent with findings showing that low levels of emotional support predict the higher intake of carbohydrates [26]. On the basis of these findings and in accordance with the framework of SBT, we expect to find a covariance between loneliness and social connectedness on the one hand and consumption of sugar on the other.

### Hypotheses

Sodas and juices contain high amounts of sugar and are highly available in western societies, easy to grab and convenient to drink. The per capita consumption of soda and juice in Norway is among the highest in the world [27]. Therefore, sugar-containing sodas and juices are likely to work well as an indicator of intake of sugar that has the ability to quickly provide glucose to the bloodstream. The main hypothesis (H^1^) of the present study is thereby that degree of loneliness and social connectedness are associated with degree of intake of sugar – in the form of sugar-containing sodas and juices. However, sugary beverages consist of more than just sugar. If the null hypothesis of H^1^ should be rejected, indicating that less socially connected people consume more soda and juice than their more socially integrated counterparts, sugar may not necessarily be the reason for the higher intake (it could be the sweet taste, the water, the citric acid, or any of the other components that sodas and juices consist of). To clarify the role of sugar in the potential relationship between social connectedness and intake of sugar-containing drinks, an additional hypothesis may be useful (H^2^): degree of social connectedness is associated with degree of intake of artificially *sweetened* sodas and juices. If the null hypotheses of both H^1^ and H^2^ should be rejected, we cannot claim that intake of sugary soda and juice related to social connectedness necessarily is an expression of attractiveness to sugar. If, however, we reject the null hypothesis of H^1^ but not the one of H^2^, indicating that less socially connected people consume more sugary drinks but not more artificially sweetened drinks, we can conclude that there is a strong indication that sugar is the key-component of these relationships. In this study, we were able to extract these variables from a huge pregnancy cohort.

## Methods

The Norwegian Mother and Child Cohort Study (MoBa) is a prospective population-based pregnancy cohort study conducted by the Norwegian Institute of Public Health [28]. In the period 1999–2008, all hospitals in Norway (except two) with more than 100 births per year invited pregnant women to participate in the study provided that they could read Norwegian. They received a postal invitation three weeks before the ultrasound examination routinely offered to all pregnant women in Norway. 38.7% of invited women consented to participate, and the cohort now includes 109 000 children, 91 000 mothers and 71 700 fathers. Blood samples were obtained from both parents during pregnancy and from mothers and children (umbilical cord) at birth. Follow-up is conducted by questionnaires at regular intervals and by linkage to national health registries. Several sub-studies are conducting additional collections of data and biological materials. The current study is based on version 7 of the quality-assured data files released for research on October 17^th^ 2012. Informed consent was obtained from each MoBa participant upon recruitment. The study was approved by The Regional Committee for Medical Research Ethics in Western Norway.

### Participants

The present study includes women participating in the MoBa study for the first time. Women who participated multiple times only provided data regarding their first participating pregnancies, and participants with diabetes type 1 were excluded. The included participants (N = 90 084) had a mean age of 29.2 years (std. deviation 4.40). Among them, 46.5% were married, 50.7% were cohabitating, and 2.8% were single. The average level of education was higher for the sample than for the general population of women aged between 16–49 years (official statistics from 2008 in brackets): compulsory school, 3.3% (28.1%); vocational school/three-year college, 34.9% (34.8%), and university/higher education, 61.7% (34.4%).The full sample has been compared with the general population of pregnant women in Norway and is described in more detail elsewhere [29].

### Measures

MoBa collects many diverse variables. Of special interest for this study were sugar-containing beverages and five factors reflecting aspects of social connectedness: loneliness, marital status, couple relationship quality, social support from others than the partner and perceived cohesion at work.

To measure the consumption of sugar-containing sodas and juices, participants were asked three questions. They were asked to report how many glasses of 1) *sugar-containing cola*, 2) *other sugar-containing soda products,* and 3) *sugar-containing juices* they had been drinking daily during the last 17 weeks. To be able to assess whether potential associations between social connectedness and sugar-containing beverages are related to attractiveness to sugar, we also included reports on how many glasses of 1) *artificially sweetened cola*, 2) *other artificially sweetened sodas*, and 3) *artificially sweetened juices* the participants had been drinking daily during the same period. A table for converting bottles and large glasses into the standardized study unit (1 glass ≈ 2cl) was given in brackets next to the questions. For the purpose of this paper, the numbers of reported units of the six groups of beverages were respectively divided into seven categories spanning from ‘0 units’ to ‘6 or more units’.

*Loneliness* was measured by a five-point scale spanning from “almost never” to “almost always” based on a single item: “Do you often feel lonesome?”

*Marital status* was measured via the following categories: “married”, “co-habiting”, “single” and ‘separated/divorced’. To group the participants that were not in a relationship, “single” and “separated/divorced” were collapsed into one category.

The full ten-item version of the *Relationship Satisfaction Scale* was used to assess the quality of the co-habiting or married participants' relationships. The scale was developed for the MoBa and based on core items used in previously developed measures of marital satisfaction and relationship quality [30]–[32]. The scale has a six-step scoring format spanning from 1, “totally agree”, to 6, “don’t agree at all”. Examples of items are as follows: “My partner and I have problems in our relationship”, “I am very happy in my relationship” and “My partner is generally very understanding”. In the current sample, the scale had a Cronbach's Alpha of 0.90. This scale has been used in previous studies [33], [34].

*Social support from other than the partner* was measured via one item: “Do you have anyone other than your husband/partner whom you can ask for advice in a difficult situation?” There were three response categories: “no”, “yes, one to two people” and “yes, more than two people”.

*Perceived cohesion at work* was measured by one statement: ‘There is a great cohesion at work’, and four response categories spanning from ‘agree’ to ‘not agree at all’. For the purpose of the present analysis, the scoring scale was reversed.

### Control variables

Eight control variables that may influence the level of intake of sodas or juices were included: participation in sports, physical strain at work, body mass index, weight related self-image, depression, age, level of education and income.

Because sport activities and other physical strain increase thirst and energy utilization, they may influence the level of soda or juice consumption. We therefore included measures for engagement in sports and other physical activities, as well as physical strain at work. *Sports* was measured by 14 items covering activities such as, “running”, “biking”, “horse riding”, “dancing”, “walking” and “fitness studio”. The respondents were asked how often they had exercised during the first 17 weeks of the current pregnancy. There were five response categories: “never”, “1–3 times per month”, “once per week”, “twice per week” and “3 times or more per week”. *Physical strain at work* was based on a single item, “My work is physically hard”, and was measured on a four-point scale spanning from “correct” to “not correct”.

Individuals with high body mass index (BMI) scores tend to have elevated carbohydrate intake in the absence of hunger compared with normal-weight subjects [35]. Thus, specific mechanisms related to overweight may account for some of the variance in the consumption of sugar-containing beverages. We therefore included BMI as a control variable. BMI scores were calculated on the basis of self-reported height and weight at the beginning of pregnancy.

Weight concerns related to self-perceptions, for example, internalization of the “thin ideal”, may impact eating behaviors and dieting [36]. We therefore included a single-item variable reflecting weight concerns motivated by self-image: *“Is it important for your self-image that you keep a certain weight?”* There were three response categories: “Yes, very important”, “Yes, somewhat important” and “No, not particularly important”.

*Depression* has been linked to a desire for sweet foods [37], [38] and may therefore explain a potential association between social interaction and consumption of sweet beverages. Depression was measured based on one item from a checklist covering a wide range of diseases and health problems. The respondents were asked to mark whether they had or had had ongoing depression during the first 17 weeks of the current pregnancy.

*Age* is associated with the level of soda consumption, with young people consuming more [39], [40]. In this study, age was measured as age in years at the point of the study.

Higher educational levels are associated with lower consumption of sugar-containing soda [39]. *Educational level* was measured by six categories, from public school to >4 years of university/college.

A low level of income has been associated with high consumption of sugary drinks [40], [41]. Here, we measured *maternal income* by dividing it into seven categories, from 1 =  no income to 7 ≥ NOK 500,000 (≈ EUR 66,000).

### Statistical analyses

All statistical analyses were conducted using the IBM SPSS Version 21. To determine the bivariate associations between the main predictor variables and the outcome variables, Pearson product moment correlations were computed. The associations between the main predictor variables and consumption of cola, soda and juice (sugar- and artificially sweetened) were tested via six separate linear multiple regression analysis, one for each type of beverage as an outcome variable. The analyses were performed with the simultaneous entry of all predictor variables, including the following control variables: participation in sports, physical strain at work, body mass index, weight related self-image, depression, age, level of education and income. Because the participants did not necessarily report on all questions concerning consumption of beverages, the different models are based on different sample sizes.

## Results

First, bivariate correlations were computed between scores for loneliness, relationship satisfaction, advice from others and cohesion at work, and the six outcome variables. Pearson product moment coefficients as well as mean scores and standard deviations of scores for consumption of soda, cola and juice are given in Table 1. The results of the bivariate correlational analysis showed that all social factors were significantly correlated with scores on all sugar-containing beverages in the predicted direction. The social factors were also significantly correlated with scores on most of the artificially sweetened beverages. All correlations were generally weak, but consistently stronger between the predictor variables and the scores for sugar-containing beverages than between the predictor variables and the scores for artificially sweetened beverages. Next, a series of regression analysis was performed (see Table 2) to develop three separate models for predicting scores for the intake of sugar-containing cola, sugar-containing sodas (other than cola) and sugar-containing juices, from scores for loneliness, marital status (co-habiting and married), relationship satisfaction, advice from others and cohesion at work. All models were statistically significant; Model 1: (dependent variable: sugar-containing sodas) F (14, 28494) = 205.395, p<.001; Model 2: (dependent variable: sugar-containing cola) F (14, 25655) = 150.331, p<.001; Model 3: (dependent variable: sugar-containing juices) F (14, 50040) = 71.502, p<.001. The models explained 7.5% (adjusted R^2^  = .075), 9.1% (adjusted R^2^  = .091), and 1.9% (adjusted R^2^  = .019) of the variance in consumption of the beverages, respectively. Results from the regression analysis showed that high levels of perceived loneliness were positively associated with high levels of intake of sugar-containing soda. Significantly negative relationships emerged between both relationship satisfaction and advice from others than partner, and sugar-containing soda. Marital status and cohesion at work were not statistically significantly associated with consumption of sugar-containing soda. As shown in Table 2, all six variables related to social interaction were significantly associated with self-reported levels of intake of sugar-containing cola. A high level of perceived loneliness was associated with high levels of cola intake. Negative associations were found between cola intake and relationship satisfaction, being married (compared with being single), cohabitating (compared with being single), the possibility of seeking advice from others than the partner, and perceived cohesion at work. Table 2 also shows that high levels of perceived loneliness were associated with high levels of consumption of sugar-containing juices. Negative associations were found between relationship satisfaction, being married (compared with being single), cohabitating (compared with being single), and perceived cohesion at work on the one hand, and consumption of sugar-containing juices on the other. In this analysis, the possibility of seeking advice from others than the partner was not significantly associated with consumption of sugar-containing juices.

**Table 1:** Mean scores, standard deviations of scores, and Pearson product moment coefficients for inter-correlations of scores for main predictor variables and consumption of soda, cola and juice.

			Sugar-sweetened			Artificially sweetened		
	Mean	SD	Soda	Cola	Juice	Soda	Cola	Juice
Loneliness	1.77	0.86	.095**	.107**	.047**	.021**	.025**	.033**
Relationship satisfaction	5.32	0.65	−.060**	−.078**	−.023**	−.031**	−.044**	−.015**
Advise from others	2.49	0.57	−.078**	−.078**	−.022**	−.035**	−.023**	−.006
Cohesion at work	3.36	0.71	−.026**	−.037**	−.016**	−.013*	−.017**	−.014*

** Correlation is significant at the 0.01 level (2 tailed).

* Correlation is significant at the 0.05 level (2 tailed).

N varies from n = 29010 (lowest) to n = 65693 (highest).

**Table 2:** Multiple regression analyses: predicted scores for consumption of soda (other than cola), cola and juices from scores for loneliness, marital status (co-habiting and married), relationship satisfaction, advice from others, and cohesion at work.

	Sugar-sweetened			Artificially sweetened		
	Soda	Cola	Juice	Soda	Cola	Juice
Loneliness	.02**	.03**	.02**	−.00	−.00	.01
Married	−.05	−.12**	−.09**	−.00	.00	−.05
Cohabitating	−.02	−.09**	−.07*	.03	.02	.01
Relationship satisfaction	−.02*	−.04**	−.01*	−.01	−.03**	−.00
Advice from others	−.03**	−.02**	−.00	−.01	.00	.01
Cohesion at work	−.01	−.02**	−.01*	−.01	−.01	−.01
Mean	0.48	0.65	1.84	0.41	1.22	0.70
SD	1.00	1.22	1.36	1.14	1.84	1.30
N	25670	28509	50055	24223	29297	26618

Results are adjusted for scores on participation in sports, physical strain at work, body mass index, weight related self-image, depression, age, level of education and income.

** Correlation is significant at the 0.01 level (2 tailed).

* Correlation is significant at the 0.05 level (2 tailed).

Finally, three equivalent analyses, with the inclusion of the same control variables as included in the models above, were conducted for artificially sweetened soda (other than cola), cola and juice as outcome variables. The models were statistically significant: Model 4: (dependent variable: artificially sweetened soda) F (14, 24208) = 74.772, p<.001; Model 5: (dependent variable: artificially sweetened cola) F (14, 29282) = 177.886, p<.001; Model 6: (dependent variable: artificially sweetened juices) F (14, 26603) = 62.250, p<.001. The models explained 4.1% (adjusted R^2^  = .041), 7.8% (adjusted R^2^  = .078), and 3.1% (adjusted R^2^  =  .031) of the variance in consumption of the three types of artificially sweetened beverages, respectively. Table 2 shows that none social factors were significantly associated with consumption of artificially sweetened soda (other than cola) or juices. A significant negative association was found between relationship satisfaction and consumption of artificially sweetened cola. No other social factors were significantly associated with consumption of artificially sweetened cola.

## Discussion

Overall, our results confirmed that perceived loneliness was associated with elevated intake of sugar in the form of soda, cola and juice. High levels of relationship satisfaction, on the other hand, was negatively associated with all three types of sugary beverages. Other aspects of social connectedness such as being married, having supportive friends, and having a sense of togetherness at work were associated with lower intake of two out of three types of sugar-containing beverages. These associations were statistically significant, even after controlling for factors such as body mass index, weight-related self-image, depression, physical activity, educational level, age and income.

Although the associations were weak, all of the five tested social factors were significantly associated with consumption of at least two types of sugar-sweetened beverages. In the regression analyses, statistically significant relationships did emerge as predicted in 14 out of 18 cases. The systematic structure of these results suggests that the associations found in this sample were not coincidental. Hence, these results support the main hypothesis of the study proposing that perceived social isolation, as well as perceived social integration, influence consumption of sugar-containing sodas and juices. The assumption that sugar is a key component of the relationship between relative social isolation and intake of sugary beverages is supported by the analyses that included artificially sweetened beverages as outcome variables. Most of the variables were significantly correlated in the bivariate analyses. However, in the regression analyses, after isolating the unique contribution of the predictor variables by controlling for scores on BMI, physical activity, weight-related self-image, depression, and demographic variables, a statistically significant association with an artificially sweetened beverage emerged in only one out of 18 cases. These results are in a striking contrast to the number of statistically significant relationships that emerged for scores on sugar-sweetened drinks. This may be interpreted as a strong indication that the associations between social connectedness and sugar-containing beverages in a great part are counted for by the sugar, and to a less extent by any of the other components that soda and juices consist of. However, these findings do not rule out the possibility that other components than sugar may also come into play. One candidate is the caffeine in cola. Caffeine is a central-nervous-system stimulant that has been shown to increase vigilance, alertness, mood, ability to concentrate and ability to solve problems [42]. As the results show, sugar-containing cola is the only variable that was significantly associated with all main predictor variables. In addition, there was a significantly negative association between high levels of relationship satisfaction and artificially sweetened cola. Moreover, the independent variables had a greater effect on sugar-containing cola than on the other dependent study variables. This may be taken as an indication that caffeine might partly count for the associations found between social connectedness and cola. However, the general pattern of the present findings indicates that sugar is the factor mainly responsible for the associations between social connectedness and intake of sugary sodas and juices.

To date, very few studies have focused on the associations between social integration and intake of sugar-sweetened beverages. One exception is a study by Cacioppo and colleagues [43], in which no significant association was found between loneliness and soda consumption. This finding was based on data from a group of 89 participants and might have suffered from insufficient statistical power. A number of studies, however, have found significant associations between social factors and food consumption. For example, in an experimental study by Baumeister and colleagues [44], it was found that social rejection caused participants to eat more sweet food than non-rejected participants. In a population-based cohort study conducted in Finland, it was found that low levels of emotional support (among women) and being single or divorced (among men) were the strongest predictors of elevated levels of high-carbohydrate food consumption [26]. Moreover, researchers have also found significant associations between loneliness and overeating [45], [46]. Thus, the present results seem to be consistent with previous research on the links between social factors and nutritional intake.

The dataset used in this study did not include information on brain metabolism or blood glucose levels. Nevertheless, Social Baseline Theory [1] provides a possible explanation for the association between loneliness and intake of sugar-containing beverages that is related to relative neuronal hypoglycemia. The theory proposes that the human brain is phylogenetically designed to utilize support from social networks to save metabolic resources when engaging in demanding mental activities. As a logical consequence, individuals who perceive themselves as socially isolated must spend more neural resources on a daily basis due to the metabolic expenses of solitary strategies for problem-solving, emotion regulation and vigilance against threats. As pointed out previously in this article, when neural metabolic activity and brain glucose utilization is high, then the rapid restoration of glucose supplies is required. Lonely people may have higher neuronal metabolic activity, which requires greater glucose supplies, and hence consume more sugar than their more socially connected counterparts. Because sodas and juices are easily accessible sources of sugar, the observed associations between relative social isolation and intake of sugar-containing beverages may reflect an expression of brain glucose regulatory mechanisms [2]. In contrast, consistent with the hypothesis that individuals profit metabolically from the energy-conserving effects of close relationships, the present results showed that perceiving ones’ romantic relationship as satisfying was significantly associated with lower levels of consumption of sugar-containing beverages. This corresponds with an fMRI based study of social regulation of the threat-related brain activity demonstrating that down-regulation of neural response was a function of marital quality [25]. Further support for the hypothesis that individuals profit metabolically from social relationships is provided by the findings that being in a relationship, having supportive friends, and experiencing cohesion at work were associated with lower levels of consumption of two out of three types of sugar-containing beverages.

Some limitations of the present study should be acknowledged. The analyses were conducted based on the expectation that social factors predict the intake of sugar-containing beverages to some degree. However, a cross-sectional design does not make it possible to determine the causal relationships between research variables. On the other hand, the fact that casual directions have been demonstrated in corresponding experimental studies makes it reasonable to interpret the present results as done above. Moreover, it is challenging to see how a weak tendency to consume soda should, for example, cause changes in marital status or cohesion at work. Another concern is that despite the inclusion of several control variables, we cannot rule out the possibility that the present results may be partly explained by confounding variables not taken into account in this study. Another limitation is that this sample consisted of women in early pregnancy. The fact that pregnancy is associated with food craving [47] suggests that the scores for consumption of sweet beverages may differ between pregnant and non-pregnant woman. It should be noted that this is a limitation in terms of the generalizability of the findings, but not a limitation in terms of the assessment of the main hypothesis. However, pregnancy may be a period when women require more social support in terms of emotion regulation and practical preparations for the future. The lack of social support during pregnancy may therefore lead to a higher degree of neural activation than would otherwise occur. The associations between social factors and sugar intake may therefore be lower in the population of non-pregnant women. Gender differences may also come into play because men, in general, report a lower level of craving for sweet foods than women [18], [48], [49]. Future studies should therefore be based on samples including men and non-pregnant women, and also include consumption of non-sweet carbohydrates as outcome variables. Another limitation is that this study was based on self-report questionnaires and the data may therefore be subject to reporting bias.

## Conclusions

The aim of this study was to test a hypothesis derived from the Social Baseline Theory that relative social isolation and social connectedness influence the level of consumption of sugar. The results from a series of regression analyses support the hypothesis by showing that loneliness and social connectedness is associated with degree of sugar consumption in the form of sugar-containing sodas and juices. This is the first time the present hypothesis has been tested in a large-scale study.

## Supporting Information

Table S1 Tables S1 to S6 show data from the multiple regression analyses additional to the information given in Table 2. This includes mean scores and standard deviations of the main predictor variables, as well as unstandardized and standardized regression coefficients, t-test values and p-values.
**Predicted scores for consumption of sugar-containing soda (other than cola) from scores for loneliness, marital status (co-habiting and married), relationship satisfaction, advice from others, and cohesion at work.** Results are adjusted for scores on participation in sports, physical strain at work, body mass index, weight related self-image, depression, age, level of education and income.
(DOCX)

Table S2 **Predicted scores for consumption of sugar-containing cola from scores for loneliness, marital status (co-habiting and married), relationship satisfaction, advice from others, and cohesion at work.** Results are adjusted for scores on participation in sports, physical strain at work, body mass index, weight related self-image, depression, age, level of education and income.
(DOCX)

Table S3 **Predicted scores for consumption of sugar-containing juice from scores for loneliness, marital status (co-habiting and married), relationship satisfaction, advice from others, and cohesion at work.** Results are adjusted for scores on participation in sports, physical strain at work, body mass index, weight related self-image, depression, age, level of education and income.
(DOCX)

Table S4 **Predicted scores for consumption of artificially sweetened soda (other than cola) from scores for loneliness, marital status (co-habiting and married), relationship satisfaction, advice from others, and cohesion at work.** Results are adjusted for scores on participation in sports, physical strain at work, body mass index, weight related self-image, depression, age, level of education and income.
(DOCX)

Table S5 **Predicted scores for consumption of artificially sweetened cola from scores for loneliness, marital status (co-habiting and married), relationship satisfaction, advice from others, and cohesion at work.** Results are adjusted for scores on participation in sports, physical strain at work, body mass index, weight related self-image, depression, age, level of education and income.
(DOCX)

Table S6 **Predicted scores for consumption of artificially sweetened juice from scores for loneliness, marital status (co-habiting and married), relationship satisfaction, advice from others, and cohesion at work.** Results are adjusted for scores on participation in sports, physical strain at work, body mass index, weight related self-image, depression, age, level of education and income.
(DOCX)
