# Supplementary material for: Loneliness, Social Integration and Consumption of Sugar-Containing Beverages: Testing the Social Baseline Theory
Source: PLoS One. 2014 Aug 8;9(8):e104421. doi: 10.1371/journal.pone.0104421 (PMC4126698; doi:10.1371/journal.pone.0104421)
Supplement: Table S1 — Tables S1 to S6 show data from the multiple regression analyses additional to the information given in Table 2. This includes mean scores and standard deviations of the main predictor variables, as well as unstandardized and standardized regression coefficients, t-test values and p-values. Predicted scores for consumption of sugar-containing soda (other than cola) from scores for loneliness, marital status (co-habiting and married), relationship satisfaction, advice from others, and cohesion at work. Results are adjusted for scores on participation in sports, physical strain at work, body mass index, weight related self-image, depression, age, level of education and income. (DOCX) [file pone.0104421.s001.docx]

**Table S3.** Multiple regression analysis: predicted scores for consumption of sugar-containing soda (other than cola) from scores for loneliness, marital status (co-habiting and married), relationship satisfaction, advice from others, and cohesion at work. Results are adjusted for scores on participation in sports, physical strain at work, body mass index, weight related self-image, depression, age, level of education and income.

| Model 1 | |  |  | Unstandardized Coefficients | | Standardized Coefficients | t | Sig. |
| --- | --- | --- | --- | --- | --- | --- | --- | --- |
|  |  | Mean | SD | B | Std. Error | Beta |  |  |
|  |  |  |  |  |  |  |  |  |
|  | Loneliness | 1.72 | .83 | .026 | .008 | .022 | 3.289 | .001 |
|  | Married | 0.47 | .50 | -.108 | .066 | -.054 | -1.631 | .103 |
|  | Cohabitating | 0.52 | .50 | -.049 | .066 | -.024 | -.745 | .456 |
|  | Relationship satisfaction | 5.35 | .62 | -.033 | .011 | -.021 | -3.114 | .002 |
|  | Advice from others | 2.52 | .55 | -.060 | .011 | -.033 | -5.296 | .000 |
|  | Cohesion at work | 3.36 | .71 | -.015 | .009 | -.010 | -1.683 | .092 |
|  |  |  |  |  |  |  |  |  |

| Dependent Variable: sugar-containing soda (other than cola)  N = 25670 |
| --- |
