# Supplementary material for: Loneliness, Social Integration and Consumption of Sugar-Containing Beverages: Testing the Social Baseline Theory
Source: PLoS One. 2014 Aug 8;9(8):e104421. doi: 10.1371/journal.pone.0104421 (PMC4126698; doi:10.1371/journal.pone.0104421)
Supplement: Table S5 — Predicted scores for consumption of artificially sweetened cola from scores for loneliness, marital status (co-habiting and married), relationship satisfaction, advice from others, and cohesion at work. Results are adjusted for scores on participation in sports, physical strain at work, body mass index, weight related self-image, depression, age, level of education and income. (DOCX) [file pone.0104421.s005.docx]

| **Table S7.** Multiple regression analysis: predicted scores for consumption of artificially sweetened cola from scores for loneliness, marital status (co-habiting and married), relationship satisfaction, advice from others, and cohesion at work. Results are adjusted for scores on participation in sports, physical strain at work, body mass index, weight related self-image, depression, age, level of education and income. |
| --- |

| Model 5 | |  |  | Unstandardized Coefficients | | Standardized Coefficients | t | Sig. |
| --- | --- | --- | --- | --- | --- | --- | --- | --- |
|  |  | Mean score | SD | B | Std. Error | Beta |  |  |
|  |  |  |  |  |  |  |  |  |
|  | Loneliness | 1.71 | .82 | -.003 | .014 | -.001 | -.202 | .840 |
|  | Married | .48 | .50 | .002 | .123 | .000 | .013 | .990 |
|  | Cohabitating | .52 | .50 | .076 | .122 | .021 | .626 | .531 |
|  | Relationship satisfaction | 5.35 | .62 | -.078 | .018 | -.026 | -4.262 | .000 |
|  | Advice from others | 2.52 | .55 | .006 | .020 | .002 | .321 | .748 |
|  | Cohesion at work | 3.36 | .70 | -.013 | .015 | -.005 | -.843 | .399 |
|  |  |  |  |  |  |  |  |  |

| Dependent Variable: Artificially sweetened cola  N = 29297 |
| --- |
